# Micronutrient intake and associated factors among school adolescent girls in Meshenti Town, Bahir Dar City Administration, Northwest Ethiopia, 2020

**DOI:** 10.1371/journal.pone.0277263

**Published:** 2022-11-29

**Authors:** Birtukan Gizachew Ayal, Yeshalem Mulugeta Demilew, Hunegnaw Almaw Derseh, Atitegeb Abera Kidie

**Affiliations:** 1 School of public health, College of Medicine and Health Sciences, Woldia University, Woldia, Ethiopia; 2 Department of Nutrition and Dietetics, School of public health, College of Medicine and Health Sciences, Bahir Dar University, Bahir Dar, Ethiopia; Institute for Advanced Sustainability Studies, GERMANY

## Abstract

**Background:**

Adolescent girls have a greater nutrient demand and their poor dietary intake is associated with micronutrient deficiencies and poor maternal outcomes. Having information on micronutrient intake inadequacy in adolescent girls is critical for promoting healthy behavior and breaking the cycle of intergenerational malnutrition. Thus, this study assessed overall micronutrient intake inadequacy and associated factors among school adolescent girls in Meshenti town of Bahir Dar City Administration, North West Ethiopia.

**Methods:**

A school-based cross-sectional study was conducted among 401 adolescent girls from February 7 to 23, 2020. A Simple random sampling technique was used to select study participants. A multiple-pass 24-hour dietary recall with portion size estimation method and recommended dietary allowance cut-off point were used to assess micronutrient intake inadequacy. Overall micronutrient intake inadequacy was measured using the mean adequacy ratio. Nutrient databases were developed by ESHA FOOD PROCESSOR version 8.1 software. Data were entered into Epi-data version 3.1 and exported to SPSS version 23 for analysis. Multivariable logistic regression was performed to identify determinants of overall micronutrient intake inadequacy and an adjusted odds ratio at a p-value of less than 0.05 was used to see the strength of statistical association.

**Results:**

The prevalence of overall micronutrient intake inadequacy was 44.4% (95% CI: 39.7%-49.6%). Early adolescent age (AOR: 2.75, 95% CI: 1.71–4.42), food-insecure household (1.74, 95%CI: 1.087–2.784), low dietary diversity score (AOR = 2.83, 95% CI: 1.35–5.92), and high peer pressure on eating and body concern (AOR = 1.853, 95% CI: 1.201–2.857) were significantly associated factors with overall micronutrient intake inadequacy.

**Conclusion:**

Findings of this study revealed that micronutrient intake inadequacy among adolescent girls was a high public health problem in the study area. Therefore, attention should be given to adolescent girls of the study area, especially the ones in the early adolescent age. Interventions should also focus on nutrition-sensitive activities to address food insecurity, a less diversified diet, and the negative impact of peer influence.

## Introduction

Adolescence is a period of life in the 10–19 years age group [1]. Around 1.2 billion adolescents are present in developing nations, making up one-fifth to one-quarter of their country’s population [2]. Adolescence is the second most critical period of physical growth in the life cycle after the first year, where 25% of total height,45% of adult bone mass, and 50% of the ideal adult weight are achieved [2, 3]. Due to this physical growth and development, with menstruation, pregnancy or lactation adolescent girls have the greatest nutrient demand and are highly susceptible to malnutrition, nutrient deficiencies, delayed sexual maturation, and slow linear growth [3, 4].

Taking into consideration of high requirements of nutrients, Food and Agricultural Organization (FAO)/WHO 2004 joint report recommend for adolescent girls daily intake of 330–400μgRE vitamin A, 40mg of vitamin C, and 1.1, 1 and 16 mg of vitamin B1, B2, B3 respectively. In addition, the RDA of calcium is 1300 mg. The daily intake of 14.4 mg zinc and 62–65.4 mg iron considering low bioavailability is required for the maintenance of the health of adolescent girls. The daily required amounts of folate and vitamin B12 are 400μg and 2.4 μg respectively for adolescent girls [5].

Poor dietary intake in adolescent girls, resulting in inadequate nutrients is associated with micronutrient deficiencies such as iron deficiency anemia, preconception nutrient deficiencies, poor maternal health, and birth outcomes. It may also be associated with a decrease in cognitive development and is considered to reduce significantly human productivity from 10% to 15% and gross domestic product up to 10% in less developed countries [6–12].

Worldwide more than 50% of adolescent girls’ intake is inadequate for iron, calcium, zinc, and folate [13]. As a result of these poor nutrient intakes and other contributing factors, malnutrition is a global threat to the health of adolescent girls and the next generation [14]. In the world, the magnitude of anemia which is a general indicator of micronutrient deficiencies has been increased from 31.6% in 2000 to 32.8% in 2016 in adolescent girls and women of reproductive age [15, 16].

In developing countries, micronutrient intake among adolescent girls in general falls below the recommendation of WHO/FAO, and the prevalence of inadequacy of most micronutrients is above 50% [17]. The prevalence of inadequate intake for vitamins ranges from 20% to 84% and from 71% to 100% for minerals [18]. Although iron intake was considered adequate, the intake was usually limited to plant sources that are low in bioavailability [18]. The prevalence of vitamin A deficiency using biochemical indicators is estimated to be 20% among 10–14 years old girls and 18% among 15–19 years old girls in low socioeconomic countries [19].

In adolescent girls, the determinant factors for the inadequacy of micronutrient intake from their diets are considered to be sociodemographic or economic factors, lack of nutrition knowledge, unfair intra-household distribution of food, parental influence on eating behaviors (culture and religion of the family), peer pressure on eating and body concern, body image perception, media exposure and meal pattern (skipping of the meal) in developing countries, though these factors were not assessed in Ethiopian context [6, 20, 21]. Thus, this study adds to the literature on factors of micronutrient intake inadequacy by assessing some of the aforementioned factors.

Despite the concern of the Ethiopian government on adolescent nutrition by developing policy, strategy, and programs [22, 23], few dietary assessment studies in the country revealed suboptimal micronutrient intake of adolescent girls compared with standard recommendations by the WHO/FAO. These studies reflected inadequate intakes for most minerals and vitamins ranging from 73% to 100% [24, 25]. Prioritizing adolescent girls’ diet and having information on micronutrient intake inadequacy and associated factors are critical to promote healthy behavior on their diet and to break the intergenerational cycle of malnutrition.

Available literature mainly focused on the prevalence of inadequacy of a particular micronutrient and are only descriptive. The overall adequacy or quality of diet consumed by adolescent girls in terms of micronutrients and determinants for inadequacy are not well assessed. Therefore, this study aimed to determine the prevalence of overall micronutrient intake inadequacy and its determinants from the diet of school adolescent girls in Meshenti town of Bahir Dar City Administration, Northwest Ethiopia.

## Method and materials

### Study setting and study design

A school-based cross-sectional study was conducted in February 2020 among school adolescent girls (10–19 years) of Meshenti town. Meshenti town is one of the rural towns of Bahir Dar City Administration. It is located around 12km in the Southern direction from Bahir Dar town, Center of Amhara region. There are two governmental schools (one primary, from grade 1 up to 8 and one secondary, from grade 9 up to 12) in the town. A total of 1430 adolescent girls attended the town schools (831 girls at primary school (starting from grade four) and 599 girls at secondary school) during the study period. The students attending these schools come from both rural and urban areas.

The major economic activities of the inhabitants in Meshenti town and its surrounding villages are agriculture in the rural area and trade in the urban area. In agriculture maize, millet, teff, barley, grass pea, and coffee are commonly produced. Also, fruits like Mango, Avocado, Guava, Orange, and Banana are produced along with Khat by using groundwater and spring water.

### Source population and study population

All adolescent girls in Meshenti town schools were the source population, whereas adolescent girls attending their education in Meshenti town schools during the study period were the study population.

### Sample size determination

The sample size was determined by using a single population proportion by taking into account the following assumptions: expected prevalence of overall micronutrient intake inadequacy as 50% (since there was no prior study in the study area), 95% level of confidence, 5% margin of error. In addition, a 10% non-response rate was considered to obtain the final sample size of 422.

### Sampling procedure

First, lists of students from primary and secondary schools of the town were obtained from each school registrar’s office with their names, age, respective grade, and address. Then adolescent girls were traced from this list. There were 1430 (831 from primary and 559 from secondary) adolescent girls from that list and those were arranged by their identification number which was used as a sampling frame. After that, the required sample of adolescent girls (422) was proportionally allocated based on the number of adolescent girls found in each school. Finally, 245 and 177 adolescent girls were selected from primary and secondary schools respectively using simple random sampling by considering the distribution of estimated sample size in each school.

### Data collection tools and procedures

Data were collected by interviewer-administered questionnaires. The questionnaire used is developed by the researchers after reviewing the related literature and it included sociodemographic/economic variables, dietary related variables, intrahousehold food allocation, knowledge on nutrients, media exposure and peer pressure on eating and body concern, body image perception, and medical conditions [4, 21, 26–30]. After study participants were selected from the schools, their household addresses were traced in schools record. Then data collectors went to the girl’s house for the interview. In each interview written consent was taken from adolescent girls and verbal consent was taken from caregivers after explaining the purpose of the study. Assent was taken from caregivers for adolescent girls below age 18. Data were collected from mothers (caregivers) and adolescent girls by six public health nutrition masters students. The household food security and food allocation status were assessed using the responses of mothers, whereas knowledge on nutrients, media exposure, body image perception, and peer pressure on eating and body concern were assessed using the responses of girls.

### Data quality control mechanisms

Initially, the English version questionnaire was translated into Amharic and then translated back into English to maintain its consistency. The data collection was supervised by two supervisors (public health professionals having a degree). Two days of training was given for data collectors regarding the aim of the study, data collection procedure, photographs of utensils for portion size estimation, and the way of approaching the study participants. Reliability of all the questioners including Body image perception, An Inventory of peer influence on children’s eating and body concern(IPIEC), Household Food Insecurity Access Scale(HFIAS) were checked from previous literature in which these were adapted, and these were reliable with Cronbach’s alpha greater than 0.7 [4, 21, 26–30]. To check the validity of the questionnaire, a pretest of the questionnaire was conducted on 5% (21) of girls who were not included in the final study in the study area. Then the understanding of girls was compared with the primary aim of the questions, and when there was a difference between what they understood and what we were looking for, consulting and discussion with experts was done on how that question could best be framed to make it clearer and contextually appropriate. Lastly, the questions were adapted through modification of wordings and rephrasing. The food portion weighting scale was calibrated at zero after each measurement to ensure validity.

Overall micronutrient intake inadequacy (Yes/No) was considered as a dependent variable. Sociodemographic/economic variables, dietary related variables, medical condition, environmental influence (media exposure, peer pressure on eating and body concern, intra-household food distribution,), and personal characteristics (knowledge on nutrients, body image perception, meal skipping habit, and food dislike habit) of adolescent girls were independent variables.

### Measurements

#### Dietary assessment/measurement of nutrient intake

An interactive, multiple-pass 24-hour dietary recall questionnaire adapted and validated for use in developing countries [29] was used for portion size estimation and to assess nutrient intakes from foods or beverages consumed by adolescent girls.

Repeated interactive 24-h dietary recall was conducted in sub-sample using the multiple-pass technique. The dietary data collection was repeated in 20% (84) of adolescents in non-consecutive day from the first interview. The recall was repeated to take in to account for the day-to-day variation in nutrient consumption of adolescent girls. The dietary data collection was not conducted on holidays or fasting days. Single day recall was conducted for the remaining study participants since, there was no significant difference in micro-nutrient intake between the first and second day dietary recall (P-value was > 0.05 in paired sample T-test for all micronutrients).

Before actual data collection inspection of the market and surveillance of twenty-one households in the study area were done to collect data on common foods eaten, cooking methods, and utensils used in the area. Photographs of equipment and food portions usually eaten at one meal were taken during surveillance. These utensils were purchased at the market. After that, each utensil and portion were taken photograph and assigned a code. Those utensils used for food serving were standardized with food portions and water using a measuring cylinder and digital food portion weighing scale. The results were expressed in terms of milliliters and grams and 100 milliliters was considered as 100 grams for beverages.

Photographs of household utensils (spoons, ladles, cups, and glasses) and food portions were used to assist the participant to recall and for the determination of portion sizes of the consumed items. Furthermore, foods commonly consumed (staple foods) in the study area during the study period were listed and the lists were read for the participant after completing dietary recall to help the participant recall any food that they forgot at first pass.

Quantities of food consumed were estimated in household measures, local estimations (like Efign (by two hands of an average adult), Lat (one hand of an average adult), Coffee breakfast…), in number (orange, banana, lemon, mango, Guava, boiled potato, and boiled egg) and pieces). Foods expressed in number were collected as large, medium, and small. The respondents were asked which utensil they used from the photographic atlas and the portion at the average by the equipment. For purchased foods like pasta, Biscuits, and beverages (soft drinks) the brand name was recorded together with the number of items consumed, and these foods were bought from the market to see the nutrient concentration from their label. For mixed dishes, the nutrient content was obtained from their recipes.

Inadequate intakes of micronutrients were estimated by the proportion of the adolescent girls with intakes that fall below the RDA (RDA cut-point method) of a particular nutrient. The inadequacy of a particular nutrient was measured using nutrient adequacy ratio (NAR) whereas, the overall micronutrient intake inadequacy (nutritional inadequacy in terms of micronutrient) was measured using mean adequacy ratio (MAR) for ten micronutrients namely vitamin A, vitamin B1, vitamin B2, vitamin B3, vitamin C, Vitamin B12, folate, calcium, iron, and zinc.

#### Dietary diversity

In addition to assessing nutrient intake, the 24-hour recall data were used to determine the dietary diversity score for the adolescents. The dietary diversity was assessed using a standard tool suggested by Food and Agricultural Organization to measure women’s dietary diversity. The food items consumed within 24 hours were categorized into ten food groups based on their nutrients: those include grains (white roots, tubers, and plantains), pulses (beans, peas, and lentils), nuts and seeds, dairy, meat (poultry and fish), eggs, dark green leafy vegetables, vitamin A-rich fruits and vegetables, other vegetables, and fruits. Finally, Dietary Diversity Scores (DDS) were created as a summary measure of dietary diversity [4].

**Wealth index of the households** was determined using the Principal Component Analysis (PCA) by considering latrine, water source, household assets, livestock, agricultural land ownership, and crop production adopted from EDHS 2016 [26].

#### Knowledge

A total of seven knowledge assessing choice questions on the source of nutrients, the benefit of nutrients, and nutrient needs of adolescent girls were prepared [21, 31].

#### Peer pressure influence

An Inventory peer influence on children’s eating and body concern (I-PIEC), a self-reported measurement tool developed by Oliver & Thelen [32] was used to assess peer influence of adolescent girls on their eating and indirectly on nutrient intake. The tool consists of three constructs called **messages** (the frequency that girls’ experienced negative messages about their bodies or eating habit), **interactions** (the frequency that adolescent girls interacted (talked, exercised, or compared their bodies) with others regarding eating habits and body issues), and **likability** (the degree to which girls believed changing body weight or shape would increase their likability by their peers or friends or boys).

The items in each construct had a five-point Likert scale which instructs the individual to answer as 1 = ‘‘never (null),”2 = ‘‘almost never (1–2 day),” 3 = ‘‘not very often (3–4 day),”4 = ‘‘sometimes (5–6 day),” and 5 = ‘‘a lot (every day)” within a week. A total of 8 items measuring message, interaction, and likability were prepared. Then the scores were added for analysis. The possible range of scores was from 8–40 points. Finally, the mean of scores was computed [33].

#### Body image perception

Body image perception was assessed using a five-point Likert scale that was adapted from the study on body image perception in university students [27]. Adolescent girls were asked: “In your opinion are you…” with five response options (“Far too thin”, “A little too thin”, “Just right”, “A little overweight”, “and Very overweight” and are you happy in your current body weight or shape (yes/no). For the analysis, the five options were re-coded into three categories (“Too thin”, “Just right”, and “Too fat”).

**Food insecurity** was measured by the Household Food Insecurity Access Scale (HFIAS) which has a nine-item scale consisting of an occurrence question followed by a frequency of occurrence question during the previous month which is a structured, standardized, and validated tool developed by the USAID funded FANTA project. The participants’ response indicated a frequency of occurrence of never, rarely (1to 2 times), sometimes (3 to 10 times), and often (>10 times) for each of the questions, over the previous 4 weeks [28].

### Operational and term definitions

#### Recommended dietary allowance (RDA)

The average daily dietary intake level that is sufficient to meet the nutrient requirement of nearly all (97 to 98%) healthy individuals in a particular life-stage and gender group. It is the goal for usual intake by an individual [34].

#### Nutrient adequacy ratio (NAR)

The ratio of subject’s nutrient intake to the requirement (RDA).

#### Mean adequacy ratio (MAR)

Is the sum of NARs for nutrients divided by the number of nutrients evaluated [35].

#### Overall micronutrient intake inadequacy

When an individual’s intake mean adequacy ratio(MAR) for ten (vitamin A, B1, B2, B3, B12, vitamin C, folate, iron, calcium, and zinc) micronutrients was less than 1(100%).

#### Inadequate intake of particular micronutrient

When daily intake value of a particular nutrient (vitamin B1, B2, B3, B12, A, C, folate, calcium, zinc, and iron) was less than its RDA or when NAR for a nutrient was less than 1, otherwise considered as adequate intake [35].

#### Dietary diversity score

Adolescent girls who consumed five and above food groups from ten food groups were considered as having a high dietary diversity score and those who consumed less than five food groups were considered as having a low dietary diversity score [4].

#### Dislike food items

People hate any food items like porridge, milk, Avocado, etc. either due to taste, odor, color, religious restriction, or feeling sick while eating that food item.

#### Meal frequency

The frequency of meal was obtained by asking the participants to identify the meals they usually had as breakfast, lunch, dinner, and snacks (morning, afternoon, or evening) within a day. Thus, number of meals they had on a recall day were counted and classified as <3 and > = 3 meals per day. Individuals skipped at least one of their usual meals were considered as meal skipper.

#### Household food security status

Household experiences none of the food insecurity (access) conditions, or just experiences worry, but rarely were considered as secured, otherwise considered as food in-secured [28].

#### Knowledge

If girls answer knowledge assessing questions correctly above the mean of the total score they were considered as having sufficient knowledge otherwise considered as having insufficient knowledge [31].

#### Media exposure

Respondents were asked how often they read a newspaper, listened to the radio, or watched television. Those who responded at least once a week were considered to be exposed to that form of media [26].

#### Body image perception

**“**one’s subjective attitude toward one’s own physical appearance. It can include both one’s own mental images of his or her body as well as the feelings one has toward his or her body” or “the way one sees his/herself, what he/she believe about his/her appearance and not how others sees her/him” [36]

#### Peer pressure influence

When the peer pressure score of adolescent girls was greater than the mean score, they were considered as having **high** peer pressure influence whereas, peer pressure score less than or equal to the mean score was considered as having **low** peer pressure influence [33].

### Data management and analysis

#### Creating nutrient the database and calculation of nutrient content of food

Nutrient values per 100 gram of each food item were primarily obtained from the Ethiopian food composition tables [37, 38]. Nutrient content of certain food items that are not part of the Ethiopian food composition tables particularly for folate and vitamin B12 were supplemented from African (Tanzanian and West African) food composition tables [39, 40]. To obtain the weight and nutrient values of purchased foods their nutrient label were used to analyze their nutrient composition. These values were fed to ESHA FOOD PROCESSOR software version 8.1 to create a nutrient database. Then, the food items in portion size obtained from the 24-hour recall were converted into their corresponding weight (into gram) manually. After that, the calculated daily intakes in grams were fed to the created nutrient database. Hence, the software calculated nutrient values for consumed portions of each food item for every individual. Results were copied to excel and exported to SPSS for analysis. The average intake of first and second day consumption was taken for repeated recalls. Paired sample T-test was conducted to know the significant difference in micronutrient intakes between the first and the second day dietary recall in repeated recall among sub-sample. P-value greater than 0.05 was considered as there was no significant difference.

Micronutrients (vitamin A, vitamin B1, vitamin B2, vitamin B3, vitamin C, Vitamin B12, folate, calcium, iron, and zinc) which remain issues globally and are highly required by adolescent girls were analyzed in this specific study [15]. Finally, nutrient intakes were compared with RDA set by WHO/FAO joint expert consultation report 2004 for identifying the prevalence of inadequate intake [5]. Inadequate intakes of micronutrients were estimated by the proportion of the adolescent girls with intakes that fall below the RDA (RDA cut-point method) of a particular nutrient.

The inadequacy of a particular micronutrient was measured using nutrient adequacy ratio (NAR) whereas, the overall micronutrient intake inadequacy (quality of diet in terms of micronutrient) was measured using mean adequacy ratio (MAR) for ten micronutrients namely vitamin A, vitamin B1, vitamin B2, vitamin B3, vitamin C, Vitamin B12, folate, calcium, iron, and zinc.

The collected data from other sections of the questionnaire (independent variables) were coded and entered into Epi data version 3.1 and exported into SPSS version 23. The data were sorted, cleaned, and analyzed using SPSS version 23.

To determine the nutrient knowledge of participants, first adolescent girls who answered the knowledge assessing questions correctly were given a score 1 and for those who did not correctly answer the question score 0 were given. After that total score of the correct answers and mean values of the knowledge score were calculated.

Principal component analysis (PCA) was used to determine the wealth status of respondents. The responses of all variables were classified into two scores. The highest score was coded as 1 and the lower score was given code 0. Assumptions of PCA were checked to carry out the wealth index score. In PCA to determine the number of components that would retain, eigenvalue-one criterion was used and those variables having a commonality value of greater than 0.5 were used to produce factor scores. Then, the score for each household on the first principal component was retained to create the wealth score. Finally, tercials of the wealth score were created to categorize households as poor, medium, and rich.

Descriptive statistics like frequency and percentage for categorical variables, mean/median, and standard deviation /interquartile range were carried out for continuous variables. For continuous variables normality was checked by using histograms, and then normally distributed data were presented as mean (SD) and non-normally distributed data were presented as median (IQR).

Bivariable logistic regression analysis was used to know the crude association between each independent variable and the outcome variable (overall micronutrient intake inadequacy) and crude odds ratio was obtained. Then, to control for possible confounding effects and to identify factors that are independently associated with overall micronutrient intake inadequacy among adolescent girls, the variables in the bivariable analysis with a p-value less than 0.25 were included in multivariable logistic regression analysis with a backward approach. The Hosmer–Lemeshow test was performed for model fitness in the final model (P = 0.945). Having a p-value less than 0.05 in multivariable logistic regression analysis was used to conclude the presence of a statistically significant association between predictor variables with the response variable. The strength of statistical association was measured by an adjusted odds ratio at a 95% confidence level. Finally, the results were presented in terms of text, frequency tables, and graphs.

### Ethical consideration

Ethical approval was obtained from the Institutional Review Board (IRB) of College of Medicine and Health Science at Bahir Dar University with protocol number (0017/2020 and assigned number 002. An official letter of permission was obtained from Bahir Dar City Administration Health Department and Meshenti kebele administration office. Finally, oral permission was obtained from school directors. Before the interview, informed written and verbal consent was obtained from adolescent girls and caregivers respectively. For those aged below 18 years old assent was taken along with permission from caregivers. The confidentiality of study participants was kept anonymous in any process of the study.

## Results

### Sociodemographic/economic characteristics of the respondents

A total of 401 adolescent girls participated in the study with a response rate of 95.02%. Approximately two-thirds 258(64.3%) of study participants were in the late adolescent (15–19 years) age group. Substantial proportions, 363 (90.5%) and 306(78.7%) of adolescent girls’ mothers and fathers respectively don’t have formal education. More than one-fourth, 122(30.4%) of the participant’s households were food insecure. One hundred four (25.9%) adolescent girls had pocket money while going to school **(Table 1).**

**Table 1:** Sociodemographic/economic characteristics of school adolescent girls in Meshenti town Bahir Dar City Administration, 2020 (n = 401).

Variables	Frequency(N)	Percentage (%)
**Age**		
Early adolescent(10–14 years)	143	35.7
Late adolescent(15–19 years)	258	64.3
**Residence**		
Rural	249	62.1
Urban	152	37.9
**Religion**		
Orthodox Christian	349	87.0
Muslim	52	13.0
**Grade level**		
Primary(4–8)	239	59.6
Secondary(9–12)	162	40.4
**Mother educational status**		
No formal education	363	90.5
Formal education	38	9.5
**Father educational status (n = 389)**		
No formal education	306	78.7
Formal education	83	21.3
**Mother occupation**		
Housewife	333	83.0
Farmer	18	4.5
Merchant	31	7.7
Government employee	11	2.7
Others(daily laborer, student)	8	2
**Father occupation(n = 389)**		
Farmer	261	67.1
Merchant	87	22.4
Government employee	32	8.2
Daily laborer	9	2.3
**Family size**		
≥ 5	308	76.8
< 5	93	23.2
**Wealth index**		
Poor	135	33.7
Medium	138	34.4
Rich	128	31.9
**Household food security status**		
Secured	279	69.6
In-secured	122	30.4
**Pocket money**		
Yes	104	25.9
No	297	74.1

### Environmental influence, personal and dietary characteristics of respondents

About 186(46.4%) adolescent girls had high peer pressure influence on eating. Generally 165(41.1%) adolescent girls had insufficient knowledge on nutrients. About 276(68.8%) adolescent girls had a habit of skipping their regular meals. Dairy products were disliked by 32(20.8%) adolescent girls. Only 58(14.5%) study participants had a high dietary diversity score **(Table 2).**

**Table 2:** Environmental influence, personal and dietary related characteristics of school adolescent girls in Meshenti town, Bahir Dar City Administration, 2020. (n = 401).

Variables	Frequency(N)	Percentage (%)
**Media exposure**		
Non–exposed	224	55.9
Exposed	177	44.1
**Peer pressure influence on eating**		
High influence	186	46.4
Low influence	215	53.6
**Body image perception**		
Too thin	77	19.2
Just right	261	65.1
Too fat	63	15.7
**Happy with current body weight**		
Yes	309	77.1
No	92	22.9
**Knowledge on nutrients**		
Sufficient knowledge	236	58.9
Insufficient knowledge	165	41.1
**Dietary diversity score**		
High	58	14.5
Low	343	85.5
**Meal frequency**		
≥ 3 meals	362	90.3
< 3 meals	39	9.7
**Skipping of meal**		
Yes	276	68.8
No	125	31.2
**Dislike any food item**		
Yes	154	38.4
No	247	61.6
**Priority for girls during food allocation**		
Yes	13	3.2
No	388	96.8
**History of Illness**		
Yes	36	9
No	365	91

**Fig 1:**
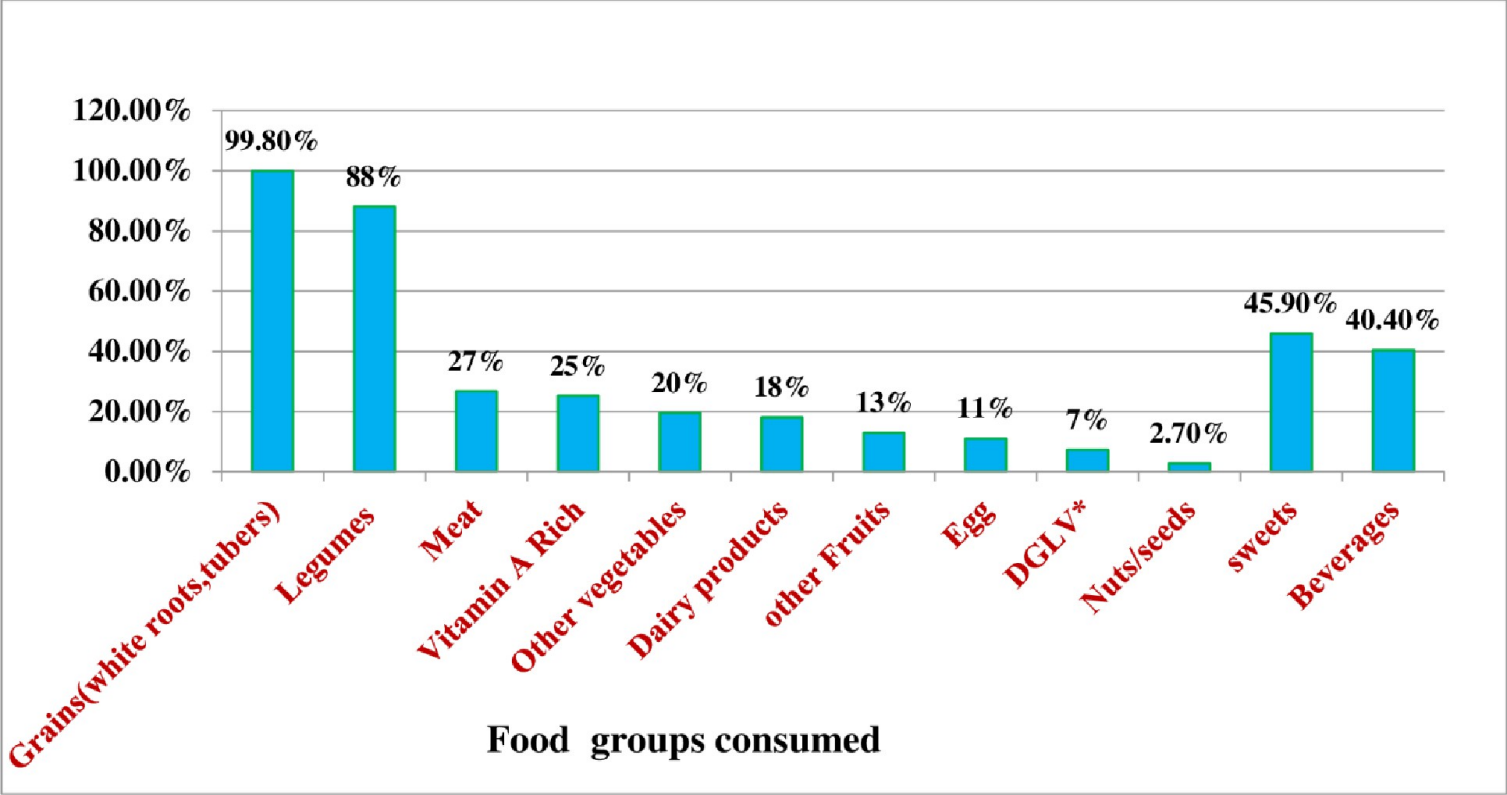
Proportion of food groups consumed by school adolescent girls in Meshenti town, 2020.

Regarding food groups consumed, plant-based foods such as cereals, roots or tubers, and legumes were the most commonly consumed food groups. Almost all, 400(99.8%) participants consumed grains (cereals, white roots, tubers) **(Fig 1)**.

### Micronutrient intake among adolescent girls

The prevalence of overall micronutrient intake inadequacy was 44.4% (95% CI: 39.7%-49.6%). The mean/median intakes of adolescent girls for micronutrients were shown in **(Table 3).**

**Table 3:** Mean (±SD) and median (IQR) of micronutrient intake of adolescent girls at Meshenti town, Bahir Dar City Administration, February 2020. (n = 401).

Nutrient	RDA*	Mean	±SD*	Median	IQR*(25th to75th)
Vitamin A(μgRE)	400			409.4	(166.42, 1042.015)
Riboflavin(vitaminB2)(mg)	1			0.62	(0.46,0.83)
Vitamin B12(μg)	2.4			1.23	(1.23, 2.14)
Ascorbic acid(mg)	40			7.85	(1.24, 33.85)
Thiamin(B1) (mg)	1.1	0.58	0.24		
Niacin(B3)(mg)	16	12.45	4.94		
Folate(μg)	400	420.61	164.66		
Iron(mg)	65.4	236.92	68.15		
Calcium(mg)	1300	597.99	262.87		
Zinc (mg)	14.4	10.57	4.003		

μg = microgram, μgRE = microgram retinol equivalent, mg = Milligram, RDA* = Recommended Dietary Allowance, SD* = Standard Deviation, IQR* = Interquartile range

**Fig 2:**
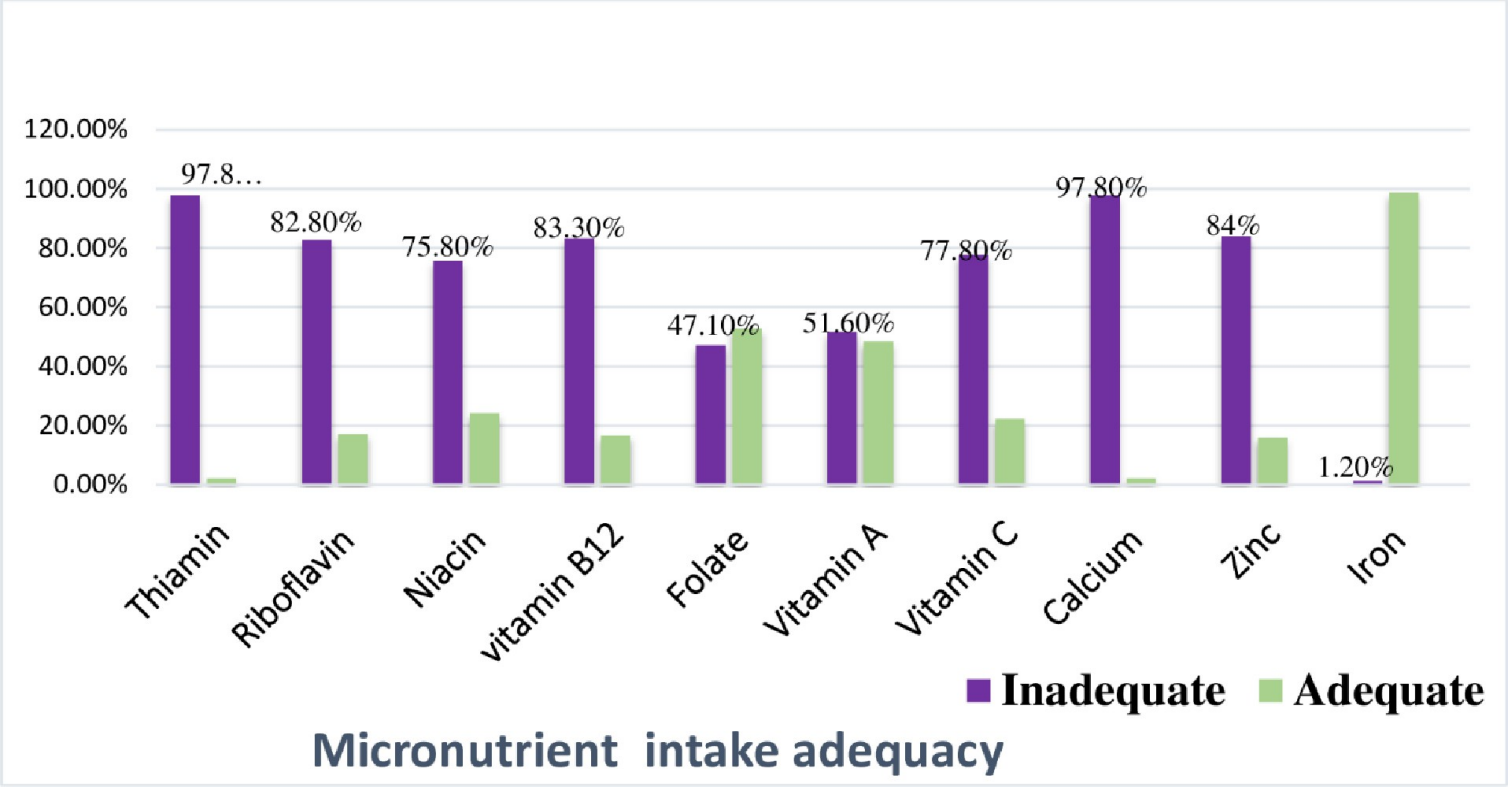
Prevalence of inadequate intake of micronutrients among school adolescent girls in Meshenti town, 2020.

The prevalence of inadequate intake for B-Vitamins was above 75% except for folate. In addition, 97.8% and 84% of adolescent girls had inadequate intake for calcium and zinc respectively. On the other hand, a low proportion, 1.2% (95%CI: 0.2%-2.5%) of adolescent girls had inadequate intake for iron. Based on age, 3.5% of early adolescent girls had inadequate intake for iron **(Fig 2).**

### Factors associated with overall micronutrient intake inadequacy among adolescent girls

In univariable binary logistic regression analysis adolescents knowledge on nutrients, having pocket money while going to school, meal frequency, adolescent age, household food security status, dietary diversity score and peer pressure on eating and body concern were factors with p-value <0.25 and were entered to multivariable analysis.

After controlling possible confounders, the result of multivariable logistic regression analysis for all the predictor variables revealed that adolescent girl’s age, peer pressure’s influence on eating and body concern, dietary diversity score, and household food security status were significantly associated with overall micronutrient intake inadequacy at p<0.05.

Adolescent girls who were in the early adolescent age group were 2.75 times more likely to had inadequate overall micronutrient intake as compared to those adolescents who were in the late adolescent age group (AOR = 2.75, 95% CI:1.71–4.42). Adolescent girls who had low dietary diversity score were 2.83 times more likely to had inadequate overall micronutrient intake compared to those who had high dietary diversity score (AOR = 2.83, 95% CI:1.35–5.92).

The odds of overall micronutrient intake inadequacy was 1.74 times higher among respondents from food in-secured households as compared to those from food secured households (AOR: 1.74, 95% CI: 1.09–2.78). Moreover, adolescent girls with high peer pressure influence were 1.85 times more likely to have inadequate overall micronutrient intake as compared to those with low peer pressure influence on eating and body concern (AOR = 1.85, 95%CI: 1.20–2.86) **(Table 4).**

**Table 4:** Factors associated with overall micronutrient intake inadequacy among school adolescent girls at Meshenti town, Bahir Dar City Administration, North West Ethiopia, 2020(n = 401).

Variables	Overall micronutrient intake inadequacy		COR (95% CI)	AOR (95%CI)
	Yes (n %)	No (n %)		
**Knowledge**				
Insufficient	82(49.7%)	83(50.3%)	1.44(0.97, 2.15)	
Sufficient	96(40.7%)	140(59.3%)	1.00	
**Meal frequency**				
< 3 meal	21(53.8%)	18(46.2%)	1.52(0.79, 2.96)	
≥ 3 meal	157(43.4%)	205(56.6%)	1.00	
**Pocket money**				
No	140(47.1%)	157(52.9%)	1.55(0.98, 2.45)	
Yes	38(36.5%)	66(63.5%)	1.00	
**Household security**				
In-secure	66(54.1%)	56(45.9%)	1.76(1.14, 2.69)	1.74(1.09, 2.78)*
Secure	112(40.1%)	167(59.9%)	1.00	1.00
**Dietary diversity**				
Low	167(48.7%)	176(51.3%)	4.05(2.03, 8.08)	2.83(1.35, 5.92)*
High	11(19.0%)	47(81.0%)	1.00	1.00
**Adolescent Age**				
10–14 years	80(55.9%)	63(44.1%)	2.07(1.37, 3.14)	2.75(1.71, 4.42)*
15–19 years	98(38%)	160(62%)	1.00	1.00
**Peer pressure**				
High	91(48.9%)	95(51.1%)	1.41(0.95, 2.09)	1.85(1.20, 2.86)*
Low	87(40.5%)	128(59.5%)	1.00	1.00

1.00: Reference group, COR: Crude Odds Ratio, AOR: Adjusted Odds Ratio, CI: Confidence Interval, *significant at p<0.05.

## Discussions

The finding of this study showed that the prevalence of overall micronutrient intake inadequacy was 44.4% (95% CI: 39.7%-49.6%) among adolescent girls. This implies that those proportions of study participants are at risk of developing micronutrient deficiency if the current dietary pattern is followed for a long period [11]. The poor micronutrient intake in this study population could happen for a number of reasons. The first reason might be due to low consumption of animal sources of food, fruits, and vegetables which are major sources of micronutrients. Low economic status might be also a barrier for accessibility micronutrient-rich foods since most of the nutrient-rich foods especially animal sources are high in price and became difficult to access [20]. Another reason might be the low educational status of parents of study participants. Because education increases the knowledge of parents on nutrition which drives dietary choices for their daughter away from sugary foods toward high-quality micronutrient food sources [20]. Moreover, the poor micronutrient intake in adolescent girls might be explained by the lack of awareness of adolescents on micronutrient needs and benefits for girls’ health [21].

Even though intake of almost all micronutrients was insufficient in the majority of adolescent girls iron intake was adequate among the majority of the girls. Only 1.2% of adolescent girls had inadequate intake of iron from their diet. The reason for this might be due to staple foods consumed in the study area that are Millet and Teff in the form of Injera (Ethiopian flat bread). These foods have a high content of iron (40.8mg/100g for Teff Injera and 37.3mg/100g for Millet Injera) [37, 38]. Thus, adolescent girls can achieve the recommended iron approximately by eating half-piece Injera. On the other hand, a higher prevalence of inadequate intake for iron was reported from Southern Ethiopia [25]. This might be due to the difference in the staple food consumed. The staple food among adolescent girls in Southern Ethiopia was unleaved bread made from unrefined maize flour which is low in iron content (8mg/100g) [38] as compared to a staple food in the present study area.

Analysis of the factors associated with overall micronutrient intake inadequacy revealed that those in the early adolescent age group were 2.75 times more likely to have inadequate micronutrient intake as compared to late adolescent age group girls. This is in agreement with studies in Spain and India [18, 41]. This might be due to early adolescent girls spent more time out of home away from caregiver supervision and might be prone to consume less nutritious food and might skip meal as reason of playing [42]. It might also be due to less awareness of their nutrient requirement. The other possible explanation might be due to unfair intrahousehold food distribution for young age than older in rural areas. However, the study finding in Brazil reported a higher prevalence of inadequate intake for iron in late adolescent girls than early adolescent girls, which is different from the present study finding [43].

In addition, this study demonstrated that dietary diversity score was negatively associated with inadequacy of micronutrient intake. The likelihood of micronutrient intake inadequacy was higher among adolescent girls who had low dietary diversity score compared to those who had high dietary diversity score. This is supported by the studies done on dietary diversity score as a measure of nutritional adequacy that showed a positive correlation between MAR and DDS [20, 44, 45]. This might be because dietary diversity is the proxy indicator of diet quality or dietary habit [4] and thus having poor dietary habits can lead to micronutrient inadequacy. In addition, there is always a marked variability in the concentration of nutrients in a single foodstuff. So, when the variety increase, nutrient content also increased; since a single foodstuff does not contain all micronutrients in the right proportion. This suggests that only a mixed or diversified diet can satisfy the nutrient requirements of adolescent girls or all age and gender groups of human beings [44].

Overall micronutrient intake inadequacy was 1.74 times higher among adolescent girls from food-insecure households as compared to those from food secured households in this study. This is comparable to the study findings done by Jun et al. and Belachew et al. [46, 47]. This is because food security measures economic access to sufficient food to meet dietary needs for healthy life and it is a predominant concern in a problem of food access. Therefore, it becomes a precondition for the daily dietary intake of adolescent girls and micronutrient intake adequacy [6, 28].

Another factor that was significantly associated with overall micronutrient intake inadequacy in this study was peer pressure’s influence on eating and body concerns. It was found that high peer pressure influence on eating and body concern increased the likelihood of inadequate overall micronutrient intake by 2 fold as compared to low peer pressure. This is supported by studies done on the effect of peer influence on eating behavior [33, 48]. This might be due to girls with high peer influence might have a concern about body weight and may like to be thin as a result e4rcc of food eaten) which could lead to the inadequacy of micronutrient intake.

Some studies have argued that nutritional knowledge had positive effect on better quality diet and changing poor dietary habit [21, 49, 50]. However, in the present study this did not hold true. Knowledge of adolescent girls on nutrients was not effective on micronutrient intake. This is consistent with previous study finding [51, 52] and suggests that giving an emphasis only on increasing the knowledge may not be the best approach for eliciting dietary behavioral change. Rather these results suggest that behavioral focus may be a necessary component for effective intervention [52].

Using 24-hour multiple pass recall, using photographs of equipment and a list of staple foods minimizes recall bias in this study. Despite these methodological strengths, the study is not free from recall bias and social desirability bias. A single-day recall might not describe the habitual intakes of an individual, though it was tried to compensate by using repeated recall in sub sample of the participants, using sufficient sample size and avoid to collect information on holidays. Food composition tables in developing countries may not reflect the local foods.

## Conclusions

The study revealed that overall micronutrient intake inadequacy was a high public health problem in the study area. Moreover, the age of adolescent girls, household food security, dietary diversity score, and peer pressure on eating and body concerns were significantly associated with the overall micronutrient intake inadequacy of adolescent girls. Therefore, attention should be given to adolescent girls of the study area, especially the ones in the early adolescent age. Schools should develop behavioral change communication activities to prevent the negative impact of peer influence. Cultivation of crops, fruits, and vegetables at the household level to improve dietary diversity and food security status are recommended. School feeding programs and supplementation of adolescent girls with micronutrients identified as inadequate should be also considered. Moreover, further analytical studies to identify other unexplored factors for micronutrient intake inadequacy in adolescent girls are recommended.

## Supporting information

S1 File (DOCX)

S1 Data (SAV)

S2 Data (SAV)

## Abbreviations

DGLV: Dark Green Leafy Vegetables
DDS: Dietary Diversity Score
EDHS: : Ethiopian Demographic and Health Survey
FANTA: Food And Nutrition Technical Assistance
FAO: Food and Agriculture Organization
HFIAS: Household Food Insecurity Access Scale
I-PIEC: Inventory Peer Influence on Children‘s Eating and body Concern
MAR: Mean Adequacy Ratio
NAR: Nutrient Adequacy Ratio
PCA: Principal Component Analysis
RDA: Recommended Dietary Allowance
SPSS: Statistical Package of Social Science
USAID: United States Agency International Development
WHO: World Health Organization
