# Supplementary material for: Micronutrient intake and associated factors among school adolescent girls in Meshenti Town, Bahir Dar City Administration, Northwest Ethiopia, 2020
Source: PLoS One. 2022 Nov 29;17(11):e0277263. doi: 10.1371/journal.pone.0277263 (PMC9707767; doi:10.1371/journal.pone.0277263)
Supplement: S1 File — (DOCX) [file pone.0277263.s001.docx]

## Annex 1 English version questioner

**BAHIR DAR UNIVERSITY**

**COLLEGE OF MEDICIENE AND HEALTH SCIENCE**

**SCHOOL OF PUBLIC HEALTH**

**Questionnaire designed to assess nutrient intake and associated factors among school adolescent girls at Meshenti town, Bahir Dar city administration, North West Ethiopia, 2020.**

**Instruction**: This questionnaire is designed for the purpose of face to face interview to collect data from adolescent girls and mothers (caregivers).

**Note**: This questionnaire has to be filled only by the interviewer once informed consent or assent is obtained from respondents. Put the answer in blank spaces for open ended questions and circle for multiple choice responses.

| **Questioner Code** | _____________________ |
| --- | --- |
| Type of school(primary or secondary) | __________________________ |
| Place of residence(Got) | ______________________ |
| Date of interview(date/month/year | ___________/______________/__________ |
| Time of interview started______________ | Time of interview ended _______________ |
| Interviewer /data collector | Name _______________________________ sign._____ |
| Checked by Supervisor | Name________________________________sign_______ |

| **Part I: Sociodemographic characteristics of the study participant(Adolescent girls)** | | | |
| --- | --- | --- | --- |
| **s. no** | **Questions** | **Responses (categories)** | **Skip** |
| 100 | How old are you?(age in complete years) | ______years |  |
| 101 | What is your religion? | 1.Orthodox Christian  2.Muslim |  |
| 102 | What grade are you? | ________________ |  |
| 103 | What is your mother/caregiver educational status? | 1.Can’t read and write  2,Can read and write  3,Primary(1-8)  4,Secondary(9-10)  5.preparatory(11-12)  6,college and above |  |
| 104 | What is your father educational status? | 1.Can’t read and write  2,Can read and write  3,Primary(1-8)  4,Secondary(9-12)  5.preparatory(11-12)  5,college and above | If no father ,write as don’t have father |
| 105 | What is the occupation of your father? | 1.Farmer  2.Merchant  3 government Employee  4.Student  5.daily laborer  6. Other (specify)__________ | If no father ,write as don’t have father |
| 106 | What is the occupation of your mother? | 1.House wife  2.Farmer  3.Employee  4.Merchant  5.daily laborer  6.Student  7.Other (specify_________ |  |
| 107 | Did your parents give money (pocket money) for buying food when you go school? | 1.yes 2.no |  |
| 108 | Family size(number of people live in your house) | _________in number |  |

**Part II: Questions for assessing house hold socio economic status (wealth index)**

| **A. Housed hold assets and housing condition** | | |
| --- | --- | --- |
| 200 | What is the condition of owner ship of the house? | 1.private  2. rental/governmental |
| 201 | What is the main construction material used for wall of house? | 1.cement,wood,mud  2.wood with mud |
| 202 | What is the main construction material used for the Roof of house? | 1. Corrugated iron/metal  2. Plastic  3.chipud/modern kornes |
| 203 | What is the main construction material used for the floor of your house? | 1.sand  2.sand with plastic cover  2.Cement |
| 204 | Did you have a separating room for sleep? | 1.yes 2.no, **if no skip to Q206** |
| 205 | If yes for Q 204 how many rooms does your house have? | ______in number |
| 206 | Can you tell me please if any member of your house have any of the following? (multiple answers are possible) | 1.Electricity  2.wall Clock  3.Television  4. Home phone  5. Refrigerator  6 Electric Mitad/stove  7 Bed with cotton, sponge mattress  8 hand watch  9 Sofa  10 Mobile  11 Radio  12 Dining table  13 Solar |
| 207 | What type of fuel does your household mainly use for cooking? (multiple answer is possible) | 1.Electricity/stove  2.charcol  3.wood  4. Animal dung  5 natural gas |
| 208 | Does any member of the household own the following? (multiple answer is possible) | 1.Bicycle  2.Motor cycle  3.Animal drawn cart  4.Car/truck  5.Bajaj |
| 209 | Does the household own any live stock or animals? | 1.yes 2.no , **if no skip to Q212** |
| 210 | If yes for Q209 how many of the following animals do you have? | A,cows/Gider/oxen/bulls \|___\|___\|__/___  B. cattle ____  C, chicken \|_______  D, goats \|________  E, sheep \|________  F, donkey/mule \|_______/_____  G, beehives \|______ |
| 211 | Did you have a separate room for animals? | 1.yes 2.no |
| 212 | Does your household own any agricultural land? | 1.yes if yes ------hectar /kada 2.no |
| 213 | Does your household own eucalyptus farmland? | 1.yes, if yes____ hectar / kada 2.no |
| 214 | How many of kuntal or kilo did you produce the following crops or fruits in a year? | 1 teff________kuntal/madaberya  2 maize_____kuntal/madaberya  3 millet______ kuntal/madaberya   1. Barley________ kuntal/madaberya 2. Nug_________ kuntal/madaberya 3. Wheat_________ kuntal/madaberya 4. orange_________ kuntal/madaberya 5. Mango______ kuntal/madaberya 6. coffee______ kuntal/madaberya |
| **B. Wash, Sanitation and Hygiene** | | |
| 215 | What is the main source of water for the member of your house hold? | 1.Pipe water  2.Public tap  3.ground water  4.well/spring  5.others(specify)_________ |
| 216 | Did you have toilet for use of your family? | 1.yes 2 no ,**if no skip to Q218** |
| 217 | If yes for Q216 What kind of toilet your households use? | 1.Traditional pit latrine  2.Pit latrine with slab  3. Ventilated improved pit latrine |
| 218 | Did you have solid waste storage/disposal material? | 1.yes 2 no |

**Part III: Questions for assessing Nutrition related (meal pattern, meal frequency, and dietary habit).**

| **S.NO** | **Questions** | **Categories or responses** | **SKIP** |
| --- | --- | --- | --- |
| **300** | How many meals do you eat within a day? | ________in number |  |
| **301** | What is your most typical meal pattern within a day? | 1. Breakfast- lunch- dinner 2. Breakfast-snack - lunch- dinner 3. Breakfast- lunch-snack- dinner 4. Breakfast-snack- lunch-snack- dinner 5. Breakfast-snack- lunch-snack- dinner- late night snack |  |
| **302** | Do you have a habit of skipping your usual meal? | 1.Yes 2.No | If no skip to Q305 |
| **303** | If yes for Q301 which meal did you usually skip? | 1.breakfast  2.lunch  3.dinner  4.snack |  |
| **304** | What is your reason to skip your meal? | 1. Tiredness/workload 2. Not to increase weight 3. No available food 4. Sickness 5. Due to fasting 6. Other (specify)_____ |  |
| **305** | Is there any food item that you dislike? | 1.Yes 2.No | **If no skip to Q308** |
| **306** | If yes for Q 305 Which food item you dislike? (multiple response is possible) | 1.Porridge,“atmit”,bread (Cereals)  2. “shiro wot”(other legumes)  3. key sir, tomato, chilly (other vegetables)  4.Egg  5. Milk, and milk products  6. Banana or other fruits  7.Meat  8.Coffee  9.Other(specify)___________ |  |
| **307** | What is the reason for your dislike? | 1. Smell/taste of food 2. Heart burn/discomfort 3. Feeling of nausea/ vomiting 4. I don’t know the reason 5. Other reason(Specify)___ |  |
| **308** | For whom does priority given in food allocation (distribution) in your household**? (Ask mothers or caregivers)** | 1.for Boys  2.for girls  3.Equal |  |

**Part IV: Dietary assessment questionnaire (24 hour dietary recall questioners for assessing nutrient intake and dietary practice of adolescent girls**

Now I would like to ask you about some liquids, solid or semi-solid food that you had yesterday during the day and night. You should recall all foods you ate yesterday both at home and outside the home. Try to recall starting from the morning. **(After recalling facilitate the recalling by reading the staple foods listed in Annex III).**

| 400. Recall(24 hr recall ) | | | | | | |
| --- | --- | --- | --- | --- | --- | --- |
| **Occasion** | Place of eaten | Food or Drink | Description and cooking method | Condition of Bowel (shared or lonely)if shared bowel try to list the number, sex and age of persons eaten together | Amount eaten (by equipment /number/ photo code | Brand name (for purchased food ) for biscuit, Saphagati, gum, alcohol, soft drinks,…. |
| Morning before breakfast |  |  |  |  |  |  |
| Breakfast |  |  |  |  |  |  |
| After breakfast |  |  |  |  |  |  |
| Lunch |  |  |  |  |  |  |
| After lunch |  |  |  |  |  |  |
| Snack |  |  |  |  |  |  |
| After snack |  |  |  |  |  |  |
| dinner |  |  |  |  |  |  |

| 401 | Was food intake unusual? | 1. Yes 2. No | If yes, skip to Q403 |
| --- | --- | --- | --- |
| 402 | If yes, how was it unusual? | 1. A feast day? 2. Market day? 3. A fasting day? 4. Other-______ |  |
| 403 | Have ever been ill in the past 2 weeks? | 1.yes 2.no | If no Skip to Q500 |
| 404 | If yes, did illness affect your appetite? | 1.yes 2.no |  |
| 405 | If yes for Q 404, how? | 1. Increase 2. Decrease |  |

**Part V: Occurrence and Frequency of Household Food Insecurity (Interview the caregivers)**

| S.no | **Questions** | **Response Option** | **Skip** |
| --- | --- | --- | --- |
| 500 | In the past four weeks, did you worry that your household would not have enough food? | 0 = No  1=Yes | If no skip to Q501 |
| 500a | How often did this happen? | 1 = Rarely (once or twice)  2 = Sometimes (3-10 times)  3 = Often (more than ten times) |  |
| 501 | In the past four weeks, were you or any household member not able to eat the kinds of foods you preferred because of a lack of resources? | 0 = No  1=Yes | If no skip to Q502 |
| 501a | How often did this happen? | 1 = Rarely (once or twice)  2 = Sometimes (3-10 times)  3 = Often (more than ten times) |  |
| 502 | In the past four weeks, did you or any household member have to eat a limited variety of foods due to a lack of resources? | 0 = No  1 = Yes | (skip to Q503 |
| 502a | How often did this happen? | 1 = Rarely (once or twice)  2 = Sometimes (3-10 times)  3 = Often (more than ten times |  |
| 503 | In the past four weeks, did you or any HH member have to eat some foods that you really did not want to eat because of a lack of resources to obtain other types of food? | 0 = No  1 = Yes | If no skip to Q504 |
| 503a | How often did this happen? | 1 = Rarely (once or twice)  2 = Sometimes (3-10 times)  3 = Often (more than ten times) |  |
| 504 | In the past four weeks, did you or any HH member have to eat a smaller meal than you felt you needed because there was not enough food? | 0 = No  1 = Yes | If no skip to Q505) |
| 504a | How often did this happen? | 1 = Rarely (once or twice)  2 = Sometimes (3-10 times )  3 = Often (more than ten times) |  |
| 505 | In the past four weeks, did you or any other HH member have to eat fewer meals in a day because there was not enough food? | 0 = No  1 = Yes | If no skip to Q506 |
| 505a | How often did this happen? | 1 = Rarely (once or twice)  2 = Sometimes (3-10 times)  3 = Often (more than ten times) |  |
| 506 | In the past four weeks, was there ever no food to eat of any kind in your HH because of lack of resources to get food? | 0 = No  1 = Yes | If no skip to Q507 |
| 506a | How often did this happen? | 1 = Rarely (once or twice)  2 = Sometimes (3-10 times)  3 = Often (more than ten times) |  |
| 507 | In the past four weeks, did you or any HH member go to sleep at night hungry because there was not enough food? | 0 = No  1 = Yes | If no skip to Q508 |
| 507a | How often did this happen? | 1 = Rarely (once or twice)  2 = Sometimes (3-10 times)  3 = Often (more than ten times) |  |
| 508 | In the past four weeks, did you or any Member go a whole day and night without eating anything because there was not enough food? | 0 = No  1 = Yes | If no skip to Q600 |
| 508a | How often did this happen? | 1 = Rarely (once or twice)  2 = Sometimes (3-10 times)  3 = Often (more than ten times) |  |

**Part VI: Questions for assessing body image perception, peer influence of the study participant (Adolescent Girl)**

| **S.no** | **Questions** | **Responses(Categories** | **Skip** |
| --- | --- | --- | --- |
| **600** | In your opinion are you_________? | 1. Too thin 2. thin 3. Just right 4. overweight 5. Very overweight |  |
| **601** | What do you want to be look like? | 1. too thin 2. thin 3. Just right 4. overweight 5. Very overweight |  |
| **602** | Are you happy with your body Weight or shape? | 1.Yes 2.No | **If yes skip to Q605** |
| **603** | If **no** for Q 602 what do you think the reason for your weight? | 1.lack of exercise  2 problem of eating  3 due to illness  4 other(specify)________ |  |
| **604** | If no for Q602 have you ever tried changing/ skipping /decrease your eating pattern due concern of your body shape? | 1.yes 2.no |  |

**Peer influence:** Respond if the occurrences of the questions are **null, 1-2 day, 3-4 day, 5-6 day and every day** within a week as never, almost never, not often, sometimes and always respectively.

| **Message** | **605** | Do your friends say you as you are fat or thin? | 1.Never  2. Almost never  3.not often  4.Sometimes  5.always |
| --- | --- | --- | --- |
|  | **606** | Do your friends say that you would go on eat less or eat more or change your usual eating? | 1.Never  2. Almost never  3.not often  4.Sometimes  5.always |
|  | **607** | Do your friends say that you ate food that will make you fat or thin | 1.Never  2. Almost never  3.not often  4.Sometimes  5.always |
|  | **608** | Do your friends say that you would look better if change your current weight? | 1.Never  2. Almost never  3.not often  4.Sometimes  5.always |
| **Interaction** | **609** | Do your friends and and you talk about what types of food make fat or thin? | 1.Never  2. Almost never  3.not often  4.Sometimes  5.always |
|  | **610** | Do your friends and you compare the size and shape of your bodies? | 1.Never  2. Almost never  3.not often  4.Sometimes  5.always |
|  | **611** | Do your friends encourage you to change your usual eating habit? | 1.Never  2. Almost never  3.not often  4.Sometimes  5.always |
| **Likability** | **612** | Do you think that having a thin body is good way for you to be liked by others/boys. | 1.Never  2. Almost never  3.not often  4.Sometimes  5.always |

**Part VII: media and Knowledge assessing questions**

| **S.no** | **Questions** | **Responses (Categories)** | **Skip** |
| --- | --- | --- | --- |
| **700** | Have you ever heard/have information about foods/ nutrients from anyone else? | 1.yes 2.no | If no skip to 703 |
| **701** | If yes where did you get the information? (Multiple answers are possible) | 1.teachers  2.Televesion,radio  3.Health professionals  4.read magazine/ newspaper  5.friends  6.other(specify)_______ |  |
| **702** | If newspaper/magazine, radio, or television is chosen in Q701 how many times you read, listen, or watch within a week? | _________times |  |
|  | Match the following nutrients with their best importance for Q703,704 and 705 | | |
| **703** | What is the main importance of carbohydrate? | A. health promoter and regulate body function  B. body builder/for growth  C. Energy provider | 0.dont know 1.know |
| **704** | What is the main importance of vitamins or minerals? |  | 0.don’t know 1.know |
| **705** | What is the main importance of proteins? |  | 0. Don’t know 1. know |
|  | Match the following nutrients with their best source of food for Q706,707 and 708 | | |
| **706** | The major sources of carbohydrates | A. fruits and vegetables  B. Meat, egg, beans, or legumes  C. Cereals, sugar, honey | 0.don’t know  1.know |
| **707** | The major sources of Vitamins & minerals |  | 0.don’t know  1.know |
| **708** | The major sources of proteins |  | 0.don’t know  1.know |
| **709** | Food intake (nutrient requirement) during adolescent age for girls should be_________? | 1.Less than adult  2.Same as adult  3.More than adult  4.Not aware/I don’t know | 0.don’t know  1.know |

**THANK YOU FOR YOUR PARTICIPATION!!!**

Annex II: Amharic version questioner

**በባህር ዳር ከተማ አስተዳደር በመሸንቲ ከተማ በሚገኙ ትምህርት ቤቶች ውስጥ የሚማሩ እድሜያቸው ከ 10_19 የሆኑ ሴት ተማሪወችን የአመጋገብ ሁኔታ እና ከሚመገቡት ምግብ የሚያገኙት ንጥረ_ምግብ በተመለከተ ለማጥናት የተዘጋጀ መጠይቅ፡፡**

**መመሪያ፡** ይህ መጠይቅ የተዘጋጀው በቃለ ምልልስ መልክ እዚህ ጥናት ላይ ከሚሳተፉ ሴት ታዳጊ ወጣቶች ላይ መረጃ ለመሰብሰብ ነው፡ ይህ መጠይቅ የተሳታፊዎቹ ፍቃደኝነት ከታወቀ በኋላ መሞላት ያለበት በጠያቂው ብቻ ነው፡፡ እባክዎን የተቀበሉዋቸውን መልሶች የያዘውን ፊደል/ቁጥር ያክቡ ወይም የመለሱትን መልስ በባዶ ቦታው ላይ ይሙሉ፡፡

| **የጥያቄ ኮድ** | _____________________ |
| --- | --- |
| ትምህርት ቤት /ተራ ቁጥር | 1 አንደኛ ደረጃ/__________________ 2 ሁለተኛ ደረጃ/_________________ |
| የመኖሪያ ቦታ (ጎጥ) | ______________________ |
| የቃለ መጠይቅ ቀን (ቀን / ወር / ዓመት) | ___________ / ____________ /2012 |
| ቃለ መጠይቁ የተጀመረበት ሰዓት | የተጀመረበት ሰዓት—————— ያለቀበት ሰዓት_______________ |
| የቃለመጠይቅ / መረጃ ሰብሳቢው | ስም _____________________________ፊርማ_____ |
| ያረጋገጠው ተቆጣጣሪ | ስም______________________________ፊርማ____________ |

| **ክፍል 1: ሀ ​​የጥናቱ ተሳታፊ ማህበራዊ ባህሪዎች (እድሜያቸው ከ 10_19 ለሆኑ ሴት ተማሪወች)** | | | |
| --- | --- | --- | --- |
| **ተቁ** | **ጥያቄዎች** | **ምላሾች (ምድቦች)** | **ዝለል** |
| 100 | ዕድሜሽ ስንት ነው? (ዕድሜዋን በሙሉ ዓመታት ውስጥ) | _____________________ዓመት |  |
| 101 | ሃይማኖትሽ ምንድን ነው? | 1.ኦርቶዶክስ ተዋህዶ ክርስቲያን  2.ሙስሊም  3 ፕሮቴስታንት   1. ካቶሊክ 2. ሌላ(ይጠቀስ)____________________ |  |
| 102 | ስንተኛ ክፍል ነሽ? | _________ |  |
| 103 | የእናትሽ /አሳዳጊሽ የትምህርት ደረጃ ምንድን ነው? | 1. ማንበብ እና መጻፍ የማትችል  2 ፣ ማንበብ እና መጻፍ የምትችል  3 ፣ አንደኛ ደረጃ (1-8)  4 ፣ ሁለተኛ ደረጃ(9-12)  5 ፣ ኮሌጅ እና ከዚያ በላይ | እናት/አሳዳጊ ከሌላት የላትም ተብሎ ይታለፍ |
| 104 | የአባትሽ የትምህርት ደረጃ ምንድን ነው? | 1. ማንበብ እና መጻፍ የማይችል  2 ፣ ማንበብ እና መጻፍ የሚችል  3 ፣ አንደኛ ደረጃ (1-8)  4 ፣ ሁለተኛ ደረጃ(9-12)  5 ፣ ኮሌጅ እና ከዚያ በላይ | አባት ከሌላት የላትም ተብሎ ይታለፍ |
| 105 | የአባትሽ ሥራ ምንድን ነው? | 1.ገበሬ  2.ነጋዴ  3 የ መንግስት ሰራተኛ  4.ተማሪ  5 የቀን ሰራተኛ  6. ሌላ (ይግለጹ_____________ | አባት ከሌላት የላትም ተብሎ ይታለፍ |
| 106 | የእናትሽ ሥራ ምንድን ነው? | 1 የቤት እመቤት  2.ገበሬ  3. የመንግስት ሰራተኛ  4.ነጋዴ  5 የቀን ሰራተኛ  6.ሌላ (ይጥቀሱ) _________) | እናት ከሌላት የላትም ተብሎ ይታለፍ |
| 107 | ትምህርት ቤት ስትሄጂ ለምግብ ተብሎ ገንዘብ ይሰጥሻል/ትይዣለሽ? | 1 አዎ 2 የለም |  |
| 108 | የቤተሰብ ብዛት (በቤትዎ ውስጥ የሚኖሩ ሰዎች ብዛት ) | _________ ቁጥር |  |

**ክፍል 2 - የቤት የሃብት ሁኔታ ለመገምገም የተዘጋጁ ጥያቄዎች (ኢኮኖሚያዊ ሁኔታን የያዘ) ወይም የሀብት መገለጫ**

| **ሀ. ቤት ውስጥ ያሉ ንብረቶችን እና የቤት ሁኔታን በተመለከተ (ለገጠርና ለከተማ）** | | | | | እለፍ |
| --- | --- | --- | --- | --- | --- |
| 200 | ቤታችሁ ባለቤትነቱ የማን ነው? | | 1. የግል  2.የኪራይ/የመንግስት/ሌላ | |  |
| 201 | የቤቱ ግድግዳ የተሰራበት ቁስ ምንድን ነው？ | | 1 ሲሚንቶ፣ጭቃና እንጨት  2 ከእንጨትእና ከጭቃ  3. ሌላ (ይግለጹ) ____________ | |  |
| 202 | የቤቱ ጣሪያ የተሰራበት የተሰራበት ቁስ ምንድን ነው？ | | 1. ቆርቆሮ  2. ማዳበሪያ  3 ችፑድ | |  |
| 203 | የቤቱ ወለል ምንድን ነው？ | | 1.አፈር/የተለቀለቀ  2 ንጣፍ  3 ሲሚንቶ | |  |
| 204 | ቤትዎ የተለየ መኛታ ክፍል አለው? | | 1. አዎ ___________________ ክፍል 2 የለም | |  |
| 205 | የሚከተሉት እቃዎች በቤት ውስጥ አለዎት?  (ከአንድ በላይ መልስ ይቻላል) | | 1. ኤሌክትሪክ መብራት  2. የግድግዳ ሰአት  3. ቴሌቪዥን  4. የቤት ስልክ  5. ማቀዝቀዣ / ፍሪጅ  6. ኤሌክትሪክ ምጣድ / ምድጃ  7. አልጋና የጥጥ/ስፖንጅ /ስፕሪንግ ፍራሽ  8 የእጅ ሰአት  9. ሶፋ  10. ሞባይል  11. ሬዲዮ  12. የመመገቢያ ጠረፔዛ  13 የፀሐይ መብራት | |  |
| 206 | ምግብ ለማብሰል በዋነኝነት ምን ትጠቀማላችሁ？  (ከአንድ በላይ መልስ ይቻላል) | | 1 ኤሌክትሪክ ምጣድ/ስቶቭ  2 ከሰል  3 እንጨት  4 ኩበት  5 ጋዝ | |  |
| 207 | ከቤተሰቡ አባላት ውስጥ የሚከተሉት እቃዎች ያሉት ይኖራል?  （አለ የሚሉትን ያክብቡ） | | 1. ብስክሌት  2.ሞተር ብስክሌት  3. በእንስሳት የሚጎተት ጋሪ  4. መኪና  5. ባጃጅ | |  |
| 208 | እንስሳት በቤትዎ ውስጥ ይገኛሉ? | | 1 አለ 2 የሉም | | የለም ካሉ ወደ ጥ ቁ 213 ይለፉ |
| 209 | አለ ካሉ የእንስሳተን ብዛት ይጥቀሱ | | ሀ፣ ላም/ጊደር/በሬ/ወይፈን________/_______/_____/——  ለ. ጥጃ_______  መ ፣ ዶሮ ___________  ሠ ፣ ፍየል _________  ረ ፣ በግ ________  ሰ ፣ አህያ/በቅሎ __________ / __________  ቀ ፣ ቀፎዎች ______________ | |  |
| 210 | ለእንስሳት የተለየ ክፍል/በረት አለዎት? | | 1. አዎ 2 የለም | |  |
| 211 | የእርስዎ ቤተሰብ የእርሻ መሬት አለው? | | 1 አለ _______ሄክታር/______ቃዳ/______ገመድ 2 የለም | |  |
| 212 | የእርስዎ ቤተሰብ የባህር ዛፍ መሬት አለው? | | 1 አለ _______ሄክታር/______ቃዳ______ገመድ 2 የለም | |  |
| 213 | በአመት ምን ያህል ኩንታል/ማዳበሪያ እህል ያመርታሉ? | | 1 ጤፍ_____________ኩንታል/ማዳበሪያ  2 በቆሎ————ኩንታል/ማዳበሪያ  3 ዳጉሳ————ኩንታል/ማዳበሪያ  4 ገብስ————ኩንታል/ማዳበሪያ  5 ኑግ——————ኩንታል/ማዳበሪያ  6 ስንዴ————ኩንታል/ማዳበሪያ  7 ብርቱካን————ኩንታል/ማዳበሪያ  8 ማንጎ ————ኩንታል/ማዳበሪያ  9 ቡና ————ኩንታል/ማዳበሪያ | |  |
| **ለ - የመጠጥ ዉሃ እና ንፅህናን በተመለከተ** | | | | |  |
| 216 | | ለመጠጥ የምትጠቀሙትን ውሃ የምታገኙት ከየት ነው？ | | 1 የቧንቧ ዉሃ  2 የጋራ ቧንቧ ዉሃ  3. የጉድጓድ ውሃ  4 ምንጭ/ወንዝ  5. ካልተከለለ ምንጭ/ወንዝ  6 ሌላ(ይጥቀሱ）—————— |  |
| 217 | | ለቤተሰብዎ የሚሆን መጸዳጃ ቤት/ ሽንት ቤት አላችሁ？ | | 1 አለ 2 የለም |  |
| 218 | | መጸዳጃ ቤት አለ ካሉ ምን ዓይነት መጸዳጃ ይጠቀማሉ? | | 1. ባህላዊ ጉድጓድ መጸዳጃ ቤት 2. መረባርብ ያለው የጉድጓድ መጸዳጃ ቤት 3. ሽታ አልባ የጉድጓድ መጸዳጃ ቤት |  |
| 219 | | የደረቅ ቆሻሻ ማጠራቀሚያ አላችሁ？ | | 1 አለ 2 የለም |  |

**ክፍል 3 - የአመጋገብ ዘይቤ ጥያቄዎች (የአመጋገብ ሁኔታ ፣ የምግብ ጊዜ እና የአመጋገብ ልምድ)**

| **ተ ቁ** | **ጥያቄዎች** | **ምድቦች ወይም ምላሾች** | **እለፍ** |
| --- | --- | --- | --- |
| **300** | በቀን ውስጥ ስንት ጊዜ ምግብ ተመግበሻል? | ________ በቁጥር |  |
| **301** | በቀን ውስጥ የሚከተሉት መደበኛ የአመጋገብ ስርአት  ምን ይመስላል? | 1 ቁርስ ፣ምሳ ፣እራት  2 ቁርስ ፣መክሰስ ፣ምሳ ፣እራት  3 ቁርስ ፣ምሳ ፣መክሰስ ፣እራት  4 ቁርስ ፣መክሰስ ፣ምሳ ፣መክሰስ ፣እራት  5 ቁርስ ፣መክሰስ ፣ምሳ ፣መክሰስ ፣እራት፣ለሊት መክሰስ |  |
| **302** | የተለመደውን የምግብ ጊዜዎን የመዝለል（የማይመገቡበት ጊዜ）ልማድ አለዎት? | 1. አዎ  2. የለኝም | መልሱ የለኝም ከሆነ ወደ ጥ.ቁ 305 ይለፉ |
| **303** | ለጥያቄ ቁ302 መልስዎ አዎ ከሆነ የትኛውን ምግብ አብዛኛውን ጊዜ ያቋርጣሉ？ | 1.ቁርስ  2.ምሳ  3.እራት  4.መክሰስ |  |
| **304** | የምግብ ጊዜዎን የሚዘሉበት /የማይመገቡበት ምክንያት ምንድን ነው? | 1. ስለሚደክመኝ /ስራ ስለሚበዛብኝ 2. ክብደቴ እዳይጨምር 3. ምግብ ስለማይኖር 4. ስለሚያመኝ 5. በጾም ምክንያት 6. ሌላ———————— |  |
| **305** | የሚጠሉት የምግብ አይነት አለ? | 1. አዎ  2. የለም | መልሱ የለም ከሆነ ወደ ጥ.ቁ **308** ይለፉ |
| **306** | መልስዎ አዎ ከሆነ የሚጠሏቸውን/ሉትን የምግብ አይነቶች/ትን ይጥቀሱ(ከአንድ በላይ መልስ ይቻላል) | 1. ገንፎ፣ አጥሚት፣ ደቦ፣ተልባ  2.ሽሮ ወጥ(ሌላ ጥራጥሬ)  3.ቀይስር፣ ቲማቲም፣ ቃሪያ (ሌሎች አትክልቶች)  4.እንቁላል  5. ወተት እና የወተት ውጤቶች  6. ሙዝና ሌሎች ፍራፍሬዎች  7. ስጋ  8. ቡና  9. ሌላ ከሆነ ይጥቀሱ ____________ |  |
| **307** | ምግቡ ስለሚያስጠላኝ ካሉ በምን ምክንያት ነው የሚያስጠላዎ? | 1. የምግቡ ሽታ (ጣዕም)  2 ደረቴን ስለሚያቃጥለኝ(ቃር)/ ምቾት ስለማይሰጠኝ/  3.ለሚያቅለሸልሸኝ/ስለሚያስመልሰኝ  4.ምክንያቱን አላውቀውም  5. ሌላ ምክንያt (ይጥቀሱ)___________ |  |
| **308** | በቤተሰብዎ ውስጥ ምግብ አቅርቦት ላይ የተሻለ/ ቅድሚያ የሚሰጠው ለማን ነው? | 1. ለሴት ልጆች  2. ለወንድ ልጆች  3 እኩል |  |

**ክፍል 4; ባለፈው 24 ሰ ዓ ት ዉስጥ እድሜያቸውከ 10_19 የሆኑ ሴት ልጆች የተመገቡአቸውን ምግቦች ለመዳሰስ የቀረቡ ጥያቄዎች**

ከዚህ በመቀጠል በትናንትናዉ እለት ቀንም ሆነ（በ24 ሰአት ውስጥ ）በቤት ውስጥም ይሁን ከውጭ የበሉትን ማንኛውንም ጠንካራ፣ፈሳሽ ወይም ለስላሳ ምግቦች ይዘርዝሩ፥ ሲናገሩ ጠዋት ላይ ከተመገቡት ምግብ ይጀምሩ።（**መልስ ሰጪዋ የጠቀሰችውን ምግብ እና መጠጥ ሁሉ ይጻፉ። መልስ ሰጪዋ ስትጨርስ ያልተጠቀሰችውን ምግብ ለማስታወስ ቅጽ 3 ላይ ያሉትን ምግቦች ያንብቡላትና የበላችውን ምግብ ይጻፉ）**

**1.የምግቦቹ ዝርዝር ፎርም**

| **ምግቡ የተበላበት**  **ጊዜ** | **የተበላበት ቦታ**  **(ቤት/ውጭ)** | **ምግብ ወይም መጠጥ** | **የምግብ ዝርዝር**  **（ምግቡ የተሰራበትን አይነት ይጥቀጹ）** | **የተበላው ብቻን /በጋራ**  **（**በጋራ ከተበላ ከስንት ሰው ጋር እንደተበላና አብረው የበሉትን ጾታና እድሜ ይጻፉ） | **ልጅቷ የበላችው መጠን በእቃ / በቁጥር ብዛ ት/ በፎቶ ኮድ** | **የምርት ስም** (ለተገዛ ምግብ) (ለስላሳ /ብስኩት/ ከረሜላ/ አልኮል፣ፓስታ፣ኩኪስ᎐᎐) |
| --- | --- | --- | --- | --- | --- | --- |
| ጠዋት ከቁርስ በፊት |  |  |  |  |  |  |
| ቁርስ |  |  |  |  |  |  |
| ከቁርስ በኋላ |  |  |  |  |  |  |
| ምሳ |  |  |  |  |  |  |
| ከምሳ በኋላ |  |  |  |  |  |  |
| መክሰስ |  |  |  |  |  |  |
| ከመክሰስበኋላ |  |  |  |  |  |  |
| እራት |  |  |  |  |  |  |
| ሌሊት |  |  |  |  |  |  |

| 401 | የነዚህ ምግቦች አመኃኀብ ያልተለመደ ነዉ? | 1. ኣዎ 2. አይደለም | መልስዎ አዎ ከሆነ ወደ ጥያቄ ቁ 403 ይለፉ |
| --- | --- | --- | --- |
| 402 | የነዚህ ምግቦች አመኃኀብ ያልተለመደ kehone endiet? | 1. ግብዣ/ ድግስቀን፣  2. የገበያቀን፣  3. የፆምቀን)  4.ሌላ(ግለፅ)_________ |  |
| 403 | ባለፉት 2 ሳምንታት ውስጥ ታምመው ያውቃሉ? | 1. አዎ 2 የለም | መልስዎ አይደለም ከሆነ ወደ ጥያቄ ቁ 500 ይለፉ |
| 404 | አዎ ከሆነ ህመሙ የምግብ ፍላጎትን ቀይሯል? | 1. አዎ 2 የለም |  |
| 405 | መልስዎ አዎ ከሆነ እንዴት? | 1. ጨምረ 2. ቀነሰ |  |

**ክፍል 5፡ የቤተሰብ የምግብ ዋስትና ሁኔታ ለመዳሰስ የተዘጋጀ መጠይቅ**

| ተ.ቁ | **ጥያቄዎች** | **መልስ(ምርጫዎች)** | **እለፊ** |
| --- | --- | --- | --- |
| 500 | ባለፈው አንድ ወር ውስጥ እርስዎ ወይም የቤተሰብዎ አባል በቤትዎ ውስጥ የምግብ እጥረት ያጋጥመናል ብሎ **ተጨንቆ** ያውቃል? | 0-አያውቅም  1-አዎ | አያውቅም ካሉ ወደ ጥያቄ 501 ይለፉ |
| 500a | መልስዎ አዎ ከሆነ ይህ ለምን ያህል ግዜ ተከስቷል? | 1- አልፎ አልፎ (**1_** **2** ጊዜ)  2- የተወሰነ ግዜ (3-10)  3- ብዙ ጊዜ (ከ10 ጊዜ በላይ) |  |
| 501 | ባለፍው እንድ ወር ውስጥ እርሰዎ ወይም ሌላ የቤተሰብ አባል በገንዘብ / በምግብ እጥረት ምክንያት **የሚፈልጉትን** ምግብ **ሳይመገቡ** ቀርተዋል? | 0-አያውቅም  1-አዎ | አያውቅም ካሉ ወደ ጥያቄ 502 ይለፉ |
| 501a | መልስዎ አዎ ከሆነ ይህ ለምን ያህል ግዜ ተከስቷል? | 1_ አልፎ አልፎ (**1_2** ጊዜ )  2- የተወሰነ ግዜ (3-10)  3- ብዙ ጊዜ(ከ10 ጊዜ በላይ) |  |
| 502 | ባለፈው አንድ ወር ውስጥ እርሶ ወይም ሌላ የቤተሰብ አባል በገንዘብ እጥረት ምክንያት የሚመገቧቸው ምግብ **አይነቶች** ቀንሰዋል? | 0-አያውቅም  1-አዎ | አያውቅም ካሉ ወደ ጥያቄ 503 ይለፉ |
| 502a | መልስዎ አዎ ከሆነ ይህ ለምን ያህል ግዜ ተከስቷል? | 1-አልፎ አልፎ (1-2 ጊዜ)  2- የተወሰነ ግዜ(3-10)  3-ብዙ ጊዜ(ከ10 ጊዜ በላይ） |  |
| 503 | ባለፈው አንድ ወር ውስጥ እርሶ ወይም ሌላ የቤተሰብ አባል በገንዘብ/በምግብ እጥረት ምክንያት **የማይፈልጉትን** የምግብ አይነት **ተመግበዋል**? | 0-አያውቅም  1-አዎ | አያውቅም ካሉ ወደ ጥያቄ 504 ይለፉ |
| 503a | መልስዎ አዎ ከሆነ ይህ ለምን ያህል ግዜ ተከስቷል? | 1- አልፎ አልፎ (1_2 ጊዜ)  2- የተወሰነ ግዜ (3-10)  3- ብዙ ጊዜ (ከ10 ግዜ በላይ) |  |
| 504 | ባለፈው አንድ ወር ውስጥ እርስዎ ወይም ሌላ የቤተሰብ አባል በገንዘብ / በምግብ እጥረት ምክንያት የሚመገቡትን የምግብ **መጠን** **ቀንሰዋል**? | 0-አያውቅም  1-አዎ | አያውቅም ካሉ ወደ ጥያቄ 505 ይለፉ |
| 504a | መልስዎ አዎ ከሆነ ይህ ለምን ያህል ግዜ ተከስቷል? | 1- አልፎ አልፎ（ 1_2 ጊዜ）  2- የተወሰነ ግዜ (3-10)  3- ብዙ ጊዜ(ከ10 ግዜ በላይ) |  |
| 505 | ባለፈው አንድ ወር ውስጥ በገንዘብ/ በምግብ እጥረት ምክንያት እርሶ ወይም ሌላ የቤተሰብ አባል በቀን የሚበሉባችው **ግዜያቶች ቀንሰዋል?** | 0-አያውቅም  1-አዎ | አያውቅም ካሉ ወደ ጥያቄ 506 ይለፉ |
| 505a | መልስዎ አዎ ከሆነ ይህ ለምን ያህል ግዜ ተከስቷል? | 1- አልፎ አልፎ (1፟_2 ጊዜ)  2- የተወሰነ ግዜ (3-10)  3- ብዙ ጊዜ (ከ10 ጊዜ በላይ) |  |
| 506 | ባለፈው አንድ ወር ውስጥ በገንዘብ/ በምግብ እጥረት ምክንያት ማንኛውም **የሚበላ ምግብ ከቤት ጠፍቶ** **ያውቃል**? | 0-አያውቅም  1-አዎ | አያውቅም ካሉ ወደ ጥያቄ 507 ይለፉ |
| 506a | መልስዎ አዎ ከሆነ ይህ ለምን ያህል ግዜ ተከስቷል? | 1- አልፎ አልፎ(1_2 ጊዜ)  2- የተወሰነ ግዜ (3-10)  3- ብዙ ጊዜ(ከ10 ግዜ በላይ) |  |
| 507 | ባለፈው አንድ ወር ውስጥ እርሶ ወይም ሌላ የቤተሰብ አባል በገንዘብ/ በምግብ እጥረት ምክኒያት **እየተራቡ** ምግብ **ሳይበሉ ተኝተው** ያውቃሉ? | 0-አያውቅም  1-አዎ | አያውቅም ካሉ ወደ ጥያቄ 508 ይለፉ |
| 507a | መልስዎ አዎ ከሆነ ይህ ለምን ያህል ግዜ ተከስቷል? | 1- አልፎ አልፎ (1_2 ጊዜ)  2- የተወሰነ ግዜ (3-10)  3- ብዙ ጊዜ(ከ10 ግዜ በላይ) |  |
| 508 | ባለፈው አንድ ወር ውስጥ ማንኛውም የቤተሰብ አባል በገንዘብ/ በምግብ እጥረት ምክኒያት እየተራቡ **ቀንና ሌሊት ሙሉ ምግብ ሳይበሉ** ቀርተው ያውቃሉ? | 0-አያውቅም  1-አዎ | አያውቅም ካሉ ወደ ጥያቄ 600 ይለፉ |
| 508a | መልስዎ አዎ ከሆነ ይህ ለምን ያህል ግዜ ተከስቷል? | 1- አልፎ አልፎ (1_2 ጊዜ)  2- የተወሰነ ግዜ (3-10)  3- ብዙ ጊዜ (ከ10 ግዜ በላይ) |  |

**ክፍል 6: እድሜያቸው ከ 10_19 የሆኑ ሴት ተማሪዎች የሰውነት አቋማቸውን እንዴት እንደሚያዩት እና አቻዎች（ እኩዮች የሚያደርሱትን ተጽንኦ የሚገልጹ ጥያቄዎች**

| **ተ ቁ** | **ጥያቄዎች** | **ምላሾች (ምድቦች）** | **ዝለል** |
| --- | --- | --- | --- |
| **600** | በሀሳብሽ ራስሽን እንዴት ታይዋለሽ（ስለሰውነት ክብደትሽ ምን ትያለሽ? | 1. በጣም ቀጭን 2. ቀጭን 3. መካከለኛ(ቀጭን ወይም ወፍራም አይደለሁም） 4. ወፍራም 5. በጣም ወፍራም |  |
| **601** | የሰውነት ኧቋምሽ ምን እንዲሆን ትፈልጊያለሽ? | 1. በጣም ቀጭን 2. ቀጭን 3. አሁን እንደሆንኩት 4. ወፍራም 5. በጣም ወፍራም |  |
| **60 2** | በሰውነት ክብደትሽ/ቅርጽሽ/አቋምሽ ደስተኛ ነሽ? | 1. አዎ  2. አይደለሁም | **መልስሽ አዎ ከሆነ ወደ ጥያቄ ቁ 605 እለፊ** |
| **603** | ለጥያቄ ቁጥር 602 መልስሽ አይደለም ከሆነ ጥሩ አቋም እንዳይኖርሽ ያደረገሽ ምክንያት ምን ሊሆን ይችላል ትያለሽ？ | 1 እንቅስቃሴ ስለማታደርጊ  2 የአመጋገብ ችግር  3 በህመም ምክንያት  4 ሌላ（ይጥቀሱ）——— |  |
| **60 4** | ለጥያቄ ቁጥር 602 መልስሽ አይደለሁም ከሆነ የሰውነት አቋምሽን ለማስተካከል የአመጋገብ ሁኔታሽን ለመቀየር / ለመዝለል /ለመቀነስ ሞክረሽ ታውቂያለሽ? | 1.አዎ  2.አላውቅም |  |

**የእኩዮች（የአቻ) ተጽዕኖ -**ለሚከተሉት ጥያቄዎች የጥያቄዎች መከሰት **በሳምንት ወይም በወር ውስጥ የማይሆን ከሆነ （ 1)** ፣ **1-2 ጊዜ የሚሆን ከሆነ （2）፣ 3-4 ቀን የሚሆን ከሆነ （3) ፣ 5-6 ቀን የሚሆን ከሆነ（ 4) እና 7 ጊዜ እና ከዛ በላይ ከሆነ ደግሞ（5)** በማለት መልስ ስጭ።

| **መልዕክት** | **605** | ጓደኞችሽ ወፍራም ወይም ቀጭን ነሽ ብለው አስተያየት ሰጠውሽ ያውቃሉ? | 1. በጭራሽ  2. አንዳንድ ጊዜ   1. የተወሰነ ጊዜ 2. ብዙውን ጊዜ 3. ሁልጊዜ |
| --- | --- | --- | --- |
|  | **606** | ጓደኞችሽ ምግብ መቀነስ፣መጨመር ወይም መቀየር እንዳለብሽ ነግረውሽ ያውቃሉ？ | 1. በጭራሽ  2. አንዳንድ ጊዜ   1. የተወሰነ ጊዜ 2. ብዙውን ጊዜ 3. ሁልጊዜ |
|  | **607** | ጓደኞችሽ የምትመገቢያቸው ምግቦች ወፍራም ወይም ቀጭን እንደሚያደርጉሽ ይነግሩሻል？ | 1 በጭራሽ  2 አንዳንድ ጊዜ  3 የተወሰነ ጊዜ  4 ብዙውን ጊዜ  5 ሁልጊዜ |
|  | **608** | ጓደኞችሽ አሁን ያለውን አቋምሽን/ክብደትሽን ብትቀይሪ ያምርብሻል ብለውሽ ያውቃሉ？ | 1. በጭራሽ  2. አንዳንድ ጊዜ   1. የተወሰነ ጊዜ 2. ብዙውን ጊዜ 3. ሁልጊዜ |
| **መስተጋብር** | **609** | እንች እና ጓደኖችሽ ምን ዓይነት ምግብ ሊያወፍር ወይም ሊያቀጥን እንደሚችል ታወራላችሁ？ | 1. በጭራሽ  2. አንዳንድ ጊዜ   1. የተወሰነ ጊዜ 2. ብዙውን ጊዜ 3. ሁልጊዜ |
|  | **610** | እንች እና ጓደኞሽ የሰውነታችሁን መጠንና ቅርፅ ታነፃፅራላችሁ？ | 1. በጭራሽ  2. አንዳንድ ጊዜ   1. የተወሰነ ጊዜ 2. ብዙውን ጊዜ 3. ሁልጊዜ |
|  | **611** | ጓደኞችሽ የተለመደው ምግብሽ እንድትቀይሪ ያበረታቱሻል? | 1. በጭራሽ  2. አንዳንድ ጊዜ  3 የተወሰነ ጊዜ  4 ብዙውን ጊዜ  5 ሁልጊዜ |
| **ተፈላጊነት** | **612** | የሰውነት ክብደተሽን（አቋምሽን መቀየርሽ） በጓደኞችሽ ለመወደድ ጥሩ አማራጭ ነው ብለሽ ታስቢያለሽ？ | 1. በጭራሽ  2. አንዳንድ ጊዜ  3 የተወሰነ ጊዜ  4 ብዙውን ጊዜ  5 ሁልጊዜ |

**ክፍል 7: እድሜያቸው ከ 10 _ 19 የሆኑ ሴት ተማሪዎች ስለ ንጥረ_ ምግብ ያላቸውን እውቀትና የመገናና ብዙሃን（የሚዲያ) ተጽዕኖን የሚዳስሱ ጥያቄዎች**

| **ተ ቁ** | **ጥያቄዎች** | **ምላሾች (ምድብ)** | **ዝለል** |
| --- | --- | --- | --- |
| **700** | ስለ ንጥረ ምግቦች ወይም ስለየተመጣጠነ ምግብ ከሌላ ከማንኛውም ሰው/ሚዲያ ሰምተሽ ታውቂያለሽ? | **1**. አዎ  2. የለም | መልስሽ የለም ከሆነ ወደ ጥያቄ ቁጥር 703 እለፊ |
| **701** | አዎ ከሆነ መረጃውን ያገኘሽው ከየት ነው?(ብዙ መልሶችን መመለስ ይቻላል) | 1. ከአስተማሪዎች  2. ቴሌቭዥን ፣ ሬዲዮ  3. ከጤና ባለሙያዎች  3.ከ መጽሔት / በራሪ ወረቀት  4. ከጓደኞቸሽ  5. ሌላ (ይግለጹ)————­­­­­­­­­­­­­­­­­­­­­­­­ |  |
| **702** | በጥያ.ቄ ቁጥር 701 ከመጽሔት ,ሬዲዮ ወይም ቴሌቪዥንን ከመረጡ በሳምንት ውስጥ ምን ያህል ጊዜ ያዳምጣሉ ወይም ይመለከታሉ? | _________ ጊዜ |  |
|  | የሚከተሉትን ንጥረ_ምግቦች በብዛት ከሚሰጡት ጥቅም አንጻር አዛምጅ(ለጥቁ 703፣704፣705) | | |
| **703** | ካርቦሃይድሬት ለምን ይጠቅማል? | 1 በሽታ ተከላካይ  2 ለእድገት ና ሰውነትን ለመገንባት  3 ሃይል እና ሙቀት ሰጭ | 0.አላውቅም  1አውቃለሁ |
| **704** | ቫይታሚኖች /ማዕድናት ያሏቸው ምግቦች ለምን ይጠቅማሉ? |  | 0.አላውቅም  1አውቃለሁ |
| **705** | ፕሮቲን（ገንቢ ) የሚባሉ ምግቦች ለምን ይጠቅማሉ? |  | 0.አላውቅም  1አውቃለሁ |
|  | የሚከተሉትን ንጥረ_ምግቦች በብዛት ከሚገኙባቸው የምግብ አይነቶች ጋር አዛምጅ(ለጥቁ 706፣707፣708) | | |
| **706** | ካርቦሃይድሬት | ሀ. አትክልትና ፍራፍሬዎች  ለ. ስጋ፣እንቁላል፣ባቄላ  መ አዝእርቶች፣ስኳር፣ማር | 0.አላውቅም  1አውቃለሁ |
| **707** | ቫይታሚን እና ማእድናት |  | 0.አላውቅም  1አውቃለሁ |
| **708** | ፕሮቲን |  | 0.አላውቅም  1አውቃለሁ |
| 709 | በኮረዳነት ዕድሜ ላይ （10_19 አመት) ያሉ ሴት ልጆች የአመጋገብ ሁኔታ (የሚያስፈልጋቸው ንጥረ ምግብ መጠን) ምን መሆን አለበት ትያለሽ? | 1 ከአዋቂዎች በታች  2 እንደ አዋቂ ሰው ተመሳሳይ  3 ከአዋቂዎች በላይ  4 አላውቅም | 0.አላውቅም  1አውቃለሁ |

**ለተሳትፎዎ ከልብ አመሰግናለሁ!!!**

Annex III: Staple foods of Meshenti town and its surrounding

| **Food group and item list (ask for consumption of food items beneath, in the**  **previous day** | **Did you**  **Eat in the**  **Previous day?**  **1.Yes**  **2.No** | **How much you eat within a day?** | **Description of the ingredients** |
| --- | --- | --- | --- |
| 1. **Cereals** |  | **Photo**  **code/slice**  **/No** |  |
| **1.Injera** |  | In piece/number |  |
| a.White Teff injera |  |  |  |
| b. Teff (50%) Maize (50%) |  |  |  |
| c. Teff (50%), Maize (25%), millet (25%) |  |  |  |
| d. Maize (50%), millet (25%) |  |  |  |
| e. Teff (25%), Maize (25%), millet (50%) |  |  |  |
| f. Millet (100%) |  |  |  |
| g. Millet (50%) & maize (50%) |  |  |  |
| **2.Bread/unleavened bread** |  | Photo/number/piece/local estimate |  |
| a. Maize flour (75%) & wheat flour (25%) |  |  |  |
| b. Millet flour (100%) |  |  |  |
| c. Teff flour (100%) |  |  |  |
| d. Maize, teff (25%), Millet (75%) |  |  |  |
| e. Maize flour (75%) & wheat flour (25%) |  |  |  |
| f.Ambasha |  |  |  |
| g. bread(wheat ) |  |  |  |
| h. water bread |  |  |  |
| i. Teff flour kita |  |  |  |
| j. Millet flour kita |  |  |  |
| k. wheat flour kita |  |  |  |
| **3.Pasta** |  | Photo/laddel/spoon | Tomato,onion,carrot,chili powder, gommen |
| **4.Macaroni** |  | Photo/ladle/spoon | Tomato,onion,carrot,chili powder,gommen |
| **5 Soup** |  | Cup photo | Pasta, Macaroni, lentil, sugar ,salt |
| **6.** **Rice** |  | Ladle/spoon | Tomato, onion, carrot |
| **7.** **porridge** |  | Photo | Chilli powder, Butter |
| a. Teff |  |  |  |
| b. Maize |  |  |  |
| c. Barley |  |  |  |
| d. Wheat |  |  |  |
| e. Aja + wheat |  |  |  |
| f. Aja atmit |  |  | Nut |
| g. Red Teff Atmit |  |  |  |
| **8.Kolo/Nifro** |  | Photo/local estimate |  |
| a. Maize kolo/nifro |  |  |  |
| b. kidney bean nifro |  |  |  |
| c. Roasted/boiled Chickpa |  |  |  |
| d. Barley kolo |  |  |  |
| e. Maize & bean nifro |  |  |  |
| **II. legumes& nuts /Pulses** |  |  |  |
| 9. Shiro wot |  | Ladle photo | Onion ,tomato,oil |
| 10.lentil split wot |  | Ladle photo | Onion ,tomato,oil |
| 11.Difin misir wot |  | Ladle photo | Onion ,tomato,oil |
| 12. pea split wot |  | Ladle photo | Onion ,tomato,oil |
| 13.Roasted bean |  | Photo |  |
| 14.Boiled bean |  | Photo |  |
| 15.Grass pea wot |  | Ladle photo |  |
| **III. roots and tubers** |  |  |  |
| 16.Potato wot |  | Ladle photo | Onion ,tomato, oil |
| 17. Boiled Potato |  | In number  -Small/medium/large |  |
| 18. Beats(key sir) |  | Ladle/spoon photo |  |
| 19.Carrot Alcha |  | Ladle/spoon photo | Onion , oil |
| **IV. Fruits** |  | Number |  |
| 20.Mango |  | Small/medium/large |  |
| 21.Avocado |  | Small/medium/large |  |
| 22.Orange |  | Small/medium/large |  |
| 23.Guva |  | Small/medium/large |  |
| 24.Papaya |  | Slice |  |
| 25.Banana |  | Small/medium/large |  |
| 26.Lemon |  | Small/medium/large |  |
| **V. vegetables** |  |  |  |
| 27.Cabbage |  | Ladle/spoon photo | Onion ,oil, green |
| 28 . Ethiopian collared green |  | Ladle/spoon photo | Onion ,oil |
| 29.Spinach |  | Ladle/spoon photo | Onion ,oil |
| 30.Cut tomato |  | Number | Onion,oil, green paper |
| 31.Tomato wot |  | Ladle/spoon photo | Onion,chilli powder,oil |
| 32 pumpkin wot |  |  | Oil, onion, chilli |
| 33 boiled pumpkin |  |  |  |
| 34.Green chilli |  | Number | Onion, oil, green paper |
| **VI. meat & fish** |  |  |  |
| 35. meat Red wot |  | Ladle/spoon photo | Onion,chill powder |
| 36. Dried meat wot |  | Ladle/spoon photo |  |
| 37.Chicken meat |  | Kind of meat /number |  |
| 38. Ox meat steamed |  | Ladle/spoon photo |  |
| 39.Sheep meat steamed |  | Ladle/spoon photo |  |
| **VII. Egg** |  |  |  |
| 40.Egg Firfir /silis |  | Ladle/spoon photo | Onion,oil,tomato |
| 41. Boiled egg |  | small/medium/large |  |
| **VIII. milk and milk products** |  |  |  |
| 42.Cow milk |  | Cup/glass photo |  |
| 43.Chease |  | Ladle/spoon photo |  |
| 44.Yoghort |  | Ladle/spoon photo |  |
| 45.whey |  | Cup/glass photo |  |
| **IX. fast foods, beverage and, Sweets** |  |  |  |
| 46.Mirinda |  | Plastic/glass bottle |  |
| 47. Coca cola |  | Plastic/glass bottle |  |
| 48.Sprite |  | Plastic/glass bottle |  |
| 49 Biscuit |  | Brand name, amount |  |
| 50 cookies |  | Brand name, amount |  |
| 51 Candy |  | Number |  |
| 52 Chewing gum |  | Number |  |
| 53.Honey |  | Spoon photo |  |
| 54. Tella (Homemade Beer) |  | Glass/tin photo |  |
| 55.Katicala |  |  |  |
| 56.kineto (fermented roasted barley with sugar) |  | Glass/tin photo |  |
| 57.Beer/woyin |  | Number |  |
| 58.Tea |  | Glass Photo |  |
| 59.Coffee |  | Cup photo |  |
